# A study of the reasons for prescribing and misuse of gabapentinoids in prison including their co-prescription with opioids and antidepressants

**DOI:** 10.1192/bjo.2021.774

**Published:** 2021-06-18

**Authors:** Anju Soni, Pamela Walters

**Affiliations:** 1 Broadmoor Hospital,West London NHS trust; 2 SLaM

## Abstract

**Aims:**

Electronic medical case files of male prisoners in a category B prison in London was studied to establish a prevalence during an 8-month period of the use of and the reasons for prescribing gabapentinoids in prison.

In addition, the prevalence of co-prescription of gabapentinoids with opioids and antidepressants was also assessed in light of the increased risk of respiratory depression resulting in death when these drugs are used in combination.

**Method:**

A retrospective, SystmOne electronic case-file based survey was undertaken searching by SNOMED CT supplemented by examination of free text, in a category B prison for males (Capacity 1500 prisoners; Average turnover of prisoners up to 6000 per year), including to establish practice standards related to the prescription of Gabapentinoids in the prison and determine the compliance with these.

**Result:**

In total, 109 cases were identified of prisoners having been prescribed gabapentinoids, pregabalin in 66 cases (61 per cent) and gabapentin in 43 cases (39 per cent). In 36 cases (33 per cent) prescriptions were for unlicensed indications. This in fact represented 50 per cent of the cases where the indications were documented. Half of the cases were co-prescribed gabapentinoids with an opioid substitute and 17% with antidepressants. Only in 22% of the cases there was documentation of discussion with the prisoner about the potential risks of co-prescribing with these medications. In 14 cases (13 per cent), prescribed gabapentinoids were diverted to other prisoners.

**Conclusion:**

For those prescribed gabapentinoids in prison, the indications for such use especially if off label should be reviewed and their use minimised where relevant.

The initiation of gabapentinoids in prison should be avoided.

For patients who are also receiving antidepressants and opioid substitutes or are abusing opiates, consideration should be given as to whether it is safe to continue on gabapentinoids, given the risks of misuse and death.

Issues raised by this study are likely to apply to other prisons, secure forensic psychiatric facilities and indeed community mental health and primary care as well.

From 1 April 2019, gabapentinoids have been classed as Class C controlled drugs in the UK.

